# A Call to Action Against Persistent Lack of Transparency in Vaccine Pricing Practices During the COVID-19 Pandemic

**DOI:** 10.5334/aogh.3898

**Published:** 2022-10-11

**Authors:** Gabriela Arguedas-Ramírez

**Affiliations:** 1 Universidad de Costa Rica, CR

**Keywords:** profit margin, therapeutics, vaccines, COVID-19, ethics, accountability, global health, colonialism

## Abstract

Lack of transparency in vaccine pricing practices is a problem that has been under discussion for a long time. To tackle this, the World Health Assembly adopted the resolution *Improving the transparency of markets for medicines, vaccines, and other health products* in 2019. However, despite the appalling effects of the current pandemic and the unequal global distribution of vaccines, the 2019 resolution has not been included as a fundamental pillar in the global health response to COVID-19.

Governments and public health agencies have provided public funding to pharmaceutical companies for research and development of new vaccines. Yet, information on pricing strategies and methodologies is still inaccessible. Furthermore, these companies are profiting from publicly funded research and development. But secrecy and opacity prevails in the pharmaceutical industry, affecting low and middle income countries.

Situating the demand for transparency, accountability and fair pricing of pharmaceutical products as a global health justice issue, I suggest an independent global observatory for accountability and transparency in the pharmaceutical global market should be created to help international organizations, governments and civil society in their quest for affordable and safe vaccines and therapeutics.

## Introduction

Lack of transparency in the negotiations of vaccine prices has been under discussion for a long time [1]. In 2019 the World Health Assembly adopted the resolution *Improving the transparency of markets for medicines, vaccines, and other health products*.^1^ This resolution indicates that “policies that influence the pricing of health products and that reduce barriers to access can be better formulated and evaluated when there is reliable, comparable, transparent and sufficiently detailed data^2^ across the value chain [2].” However, as Mèdecins sans Frontières [3] had alerted, that the final text of this resolution was watered down, limiting its impact in lowering pharmaceutical prices. Now, in the middle of global health crisis, we are far from equitable access to vaccines.

New vaccine pricing is a complicated process because it can be difficult to quantify costs (manufacturing, distribution, and research and development) [4]. However, in the case of all COVID-19 vaccines, governments, public health and scientific agencies have provided public funding for research and development, paying up front to give pharmaceutical companies security and lowering most of the risks usually taken by these companies when producing new vaccines. Therefore, those calculations should be less difficult to make and should be made public, as part of the accountability due to taxpayers.

## Vaccines and profits in times of global despair

In 2021 [5] the Access to COVID-19 Tools Accelerator (ACT-A) announced its new strategic plan “to address crucial gaps in access to COVID-19 tests, treatments, vaccines and personal protective equipment in low- and middle-income countries, using the latest epidemiological, supply and market information.”

The purpose of this plan is to “help prevent at least 5 million potential additional deaths, save the global economy more than US$ 5.3 trillion, and accelerate the end of the pandemic everywhere.” For this, ACT-A needs $23.4 billion by September 2022. Surprisingly, this strategy does not mention the 2019 WHO resolution nor any kind of mechanism to prevent escalating vaccines prices. There are no regulations or incentives for pharmaceutical companies to reduce their profit margins during a global health emergency.

The history of public health demonstrates that vaccination is one of the most cost-effective public health interventions and it is the most effective way to prevent increasing mortality and morbidity associated to COVID-19. However, there are substantial obstacles in the way. For instance, vaccine nationalism [6] is keeping negotiations with the pharmaceutical industry behind closed doors, thus undermining global efforts to ensure fair access to vaccines for everyone.

Light and Lexchin (2021) [7] argue that “contrary to the ethics of vaccines as a public health good, companies have kept manufacturing costs for all vaccines secret, (…) government agencies such as the Government Accountability Office (USA), National Audit Office (UK) and the Court of Auditors (EU) could produce far more accurate estimates of vaccine costs. To date, governments have not taken this step, and manufacturers are being allowed to keep their costs secret on these global public health goods” (p. 502).

Since January 2021 [8] leaks about vaccine prices have drawn attention to how big pharmaceutical companies’ usually offer the best deals to the more powerful buyers. For instance, South Africa’s government paid more than twice what the European Union did for the AstraZeneca vaccine. But this is just one of many issues. This information wouldn’t have reached media outlets if it wasn’t for accidental revelations [9] made by European government officials. Governments keep this kind of information confidential in exchange for discounts. However, most governments accept those discounts without knowing the terms and conditions offered by pharma companies to other countries. The South African government was told that $5.25 was the set price of AstraZeneca vaccine for upper-middle income countries. However, the European Union paid $2.15.

The persistent opacity and lack of effective instruments to control prices of pharmaceutical products disproportionally affects the Global South. It is ethically unacceptable that international organizations such as WHO and ACT-A have not effectively addressed this issue, knowing that lack of access translates into thousands and even millions of lives lost. Besides, there is another ethical problem regarding how pharmaceutical companies decide COVID-19 vaccines prices. These companies are profiting [10] from publicly funded [11] research and development. In fact, the discussion about private profiting from publicly funded scientific research have been going on for many years now, without reaching any concrete solution [12].

Without the significant financial, technological, and regulatory support from the US and EU governments, and without COVAX [13] channeling private funds, it would have been impossible to develop several safe and effective vaccines so soon. But secrecy and opacity prevails in the pharmaceutical industry, due to the dominant decision-making process based on market logics.

This lack of transparency is also a concern regarding negotiating terms and practices. For instance, representatives from two Latin American countries have reported, (under anonymity due to the confidentiality agreement), that Pfizer’s negotiators required “more than the usual indemnity against civil claims filed by citizens who suffer serious adverse events after being inoculated. They said Pfizer also insisted the governments cover the potential costs of civil cases brought as a result of Pfizer’s own acts of negligence, fraud, or malice. In Argentina and Brazil, Pfizer asked for sovereign assets to be put up as collateral for any future legal costs [14].”

These negotiating practices are set not only in unequal terms, but under the extreme pressure of a pandemic that has intensified global health inequities. Meanwhile, high income countries are failing to support the COVAX [15] mechanism, thus contributing to the transformation of a global health crisis into a geopolitical conundrum where some powerful countries easily take political advantage from the desperation of smaller and poorer nations [16].

Even more troubling are the ethical issues concerning ACT-A governance. Moon et al (2021) evaluation of ACT-A identified these problems: 1) the roles of the participants in the ACT-A decision making process are unclear, making accountability difficult to accomplish; 2) there is an inconsistent legal provision for information transparency; and 3) governments have an irrelevant role in ACT-A, raising concerns about political legitimacy [17].

## A call for transparency and fairness in vaccines pricing practices

Big pharmaceutical business model is based on the economic principle of profit maximization in the short run. This means that pharmaceutical and biotech companies will prioritize those markets that offer a good chance of major commercial success. Given the way incentives work in this business model, orphan drugs and neglected diseases are the direct and foreseeable consequence. It is not coherent with their business model to invest in diseases that affect mostly people who cannot pay the highest price set by the company [18]. It is relevant to note that the pharmaceutical business model based on profit maximization relates to a wide range of serious healthcare issues, such as antibiotic resistance [19] due to contradictory incentives. Public health authorities and healthcare services are promoting rational use of antibiotics, while pharmaceutical companies push to increase sales to maximize profits.

Control and regulation of prices in the pharmaceutical market is a sensitive topic because it is fundamentally at odds with the hegemonic market economy paradigm. In the US, for instance, there has been a long-standing lobby in favor of de-regulation of commercial activities [20], while at the same time, industry push for maximalist intellectual property provisions via bilateral and multilateral free trade agreements, such as the Trade Related Intellectual Rights Agreement (TRIPS) and TRIPS-plus [21]. These intellectual property provisions add more opaqueness on costs of research and development and give industry an excessive power in determining the final price of vaccines, therapeutics, and diagnostics. For this reason, several countries in the Global South had called for a patent waiver [22]. This initiative gathered support from a wider international community [23]. A less comprehensive version of the waiver proposal was approved at the World Trade Organization in June, 2022. However, “the agreement (…) is arriving far too late and is far too modest in scope to meaningfully affect global vaccine supply [24].”

As a result of these structural conditions and given the lack of political will to intervene, most LMIC have had very limited access to vaccines. Some of those countries won’t be able to vaccinate most of their citizens until 2023 [25]. Given the state of global health inequality and WHO modest leadership in matters of global health emergencies during the last few years, it is certainly not surprising that COVAX aspirations were so insufficient (covering just 20% of the population of each participant country without including any criteria of justice in allocation) [26].

In May, 2022, the Working Group on Strengthening WHO Preparedness and Response to Health Emergencies presented its report [27] to the WHO General Assembly. This is a key moment to re-address the issue of transparency in vaccines pricing practices, in a way that effectively rectifies past mistakes and omissions. In this report, equitable access to health products is define as availability “in a timely manner, accessible, affordable, acceptable, quality assured, safe, and effective for those who need them without differences among groups of people.” Consequently, a basic ethical and technical standard that delimit what is an acceptable profit margin for pharmaceutical companies, in the context of global health crisis such as COVID-19, should be included in the definition of “equitable access.”

## A first step: A global collaborative observatory for accountability and transparency in the pharmaceutical market

There are many lessons the global health community should learn from the COVID-19 pandemic. One of those lessons is that it is not possible to control a pandemic if access to life saving pharmaceutical products and other healthcare related technologies is subjected to market-oriented principles. In order to secure pharmaceutical products at affordable prices during a global health crisis, access to information on vaccines and therapeutics production costs, sales and profit margins is indispensable. However, big pharmaceutical companies have opposed and obstruct the debate on prices control of pharmaceutical products for a long time [28]. Nevertheless, there is mounting evidence that demonstrate the necessity of a better regulatory framework to control prices of medicines [29].

This pandemic has demonstrated it is time to put into action the declaration *Improving the transparency of markets for medicines, vaccines, and other health products* in a way that avoid the intense lobbying capacity of the big pharmaceutical industry. I consider that an independent global observatory for accountability and transparency in the global pharmaceutical market will help international organizations, governments and civil society in their quest for affordable and safe vaccines and therapeutics. Additionally, this observatory may contribute in the developing of a new culture of accountability in the pharmaceutical sector.

## Footnotes

There a few efforts to put into practice this declaration. One of the most notable is the French Observatory for transparency in drug policies (OTMeds), funded by former vice-president and former president of Act Up-Paris, Pauline Londeix and Jérôme Martin, in June 2019. https://otmeds.org/about/.

The resolution includes data such as information on net prices of health products, dissemination of human subject clinical trial’s results and costs, reports on sales revenues, prices, units sold, marketing costs and subsidies and incentives, patent status and marketing approval status of health products.
